# Relaxin—when a successful super-drug is failing

**DOI:** 10.1007/s00424-022-02734-3

**Published:** 2022-07-29

**Authors:** Klaus-Dieter Schlüter

**Affiliations:** Physiologisches Institut, Justus-Liebig-University Giessen, Aulweg 129, D-35392 Giessen, Germany

In this issue of *Pflügers Archiv – European Journal of Physiology*, Kowalleck and colleagues [1] report on the failure of relaxin to prevent development of hypoxia-induced pulmonary edema in rats. Relaxin has extensively been investigated in the past and was shown to exert multiple beneficial cardiovascular effects against hypertension, atrial fibrillation, heart failure, reperfusion injury, inflammation, and fibrosis. Nearly all of these different aspects of heart failure are positively affected by relaxin. Subsequently, relaxin was introduced in clinical studies. Extended phase III clinical trials show that a 48-h infusion with relaxin improved 180-day mortality in patients with acute heart failure [2]. Unfortunately, we still do not know the mechanisms by which relaxin exerts all these beneficial effects on various cell types. What is known is that relaxin binds to a definitive receptor, RXPF1, and that activation of this receptor is able to modify the activity of protein kinases, such as cAMP-dependent protein kinase (PKA), cGMP-dependent protein kinase (PKG), and mitogen-activated protein kinases (MAPK). However, activation of such a pathway is not always protective and beneficial in heart function.

Pulmonary edema might occur after a rapid ascent to a high altitude. It is a non-cardiogenic edema caused by elevated pulmonary capillary pressure. In the lung, hypoxia causes pulmonary vasoconstriction, a phenomenon known as the Euler-Liljestrand mechanism. To understand this mechanism is important to develop therapeutic options for populations living at high altitudes such as in Tibet or Kyrgyzstan [3]. The authors suggested that relaxin will counteract hypoxia-induced pulmonary injury via vasodilatation and improvement of left ventricular function. However, relaxin failed to produce such an effect. The authors make an important point: due to the different actions of the hormone, it is rather difficult to predict the final concentration. As relaxin shows a bell-shaped concentration–response curve, it is difficult to achieve a therapeutic relevant concentration. Among different complications, it is noteworthy to mention that vasodilatation caused by relaxin will change renal perfusion and filtration and increased filtration causes hypovolemia and thereby increasing the concentration of relaxin. This highlights the importance of the cardio-renal interaction that has to be considered whenever vasodilating drugs are used. Although relaxin caused vasodilatation, it caused overperfusion of the lungs that, together with an aggravated hydrostatic pressure due to gravity effects, accelerated rather than improved the edema. Furthermore, the authors observed different responsiveness of the right and left ventricles to relaxin, leading to an imbalance between both ventricles. Normally, the Frank-Starling mechanism should normalize this effect and it remained open why this did not occur in this particular experiment. However, the different responsiveness of left and right ventricles due to different hemodynamic loads has occasionally been observed and requires a proper but different treatment regime for both ventricles [4].

In conclusion, the study shows that every drug has to be carefully studied under quite different conditions specifically as long as the precise molecular mechanism is unclear. The work with angiotensin receptor type 2 (AT2) receptor–deficient mice suggests that up-regulation of this receptor is required for at least anti-fibrotic effects of relaxin. In pulmonary edema, this up-regulation may not be the case, and therefore, relaxin is less efficient. Therefore, although we lost the hope that relaxin improves pulmonary edema under these conditions, we learned a lot about the potential limitation of a former wonder drug that seemed to solve nearly all aspects of cardiovascular diseases. Overall, we need to study inter-organ interactions in the future with more intensity and this requires the analysis of complex animal experiments as performed in the study by Kowalleck and colleagues [1].

## Data Availability

Not applicable.
